# Sustained Maximal Voluntary Contraction Produces Independent Changes in Human Motor Axons and the Muscle They Innervate

**DOI:** 10.1371/journal.pone.0091754

**Published:** 2014-03-12

**Authors:** David A. Milder, Emily J. Sutherland, Simon C. Gandevia, Penelope A. McNulty

**Affiliations:** Neuroscience Research Australia, Sydney and University of New South Wales, Sydney, Australia; University of Sydney, Australia

## Abstract

The repetitive discharges required to produce a sustained muscle contraction results in activity-dependent hyperpolarization of the motor axons and a reduction in the force-generating capacity of the muscle. We investigated the relationship between these changes in the adductor pollicis muscle and the motor axons of its ulnar nerve supply, and the reproducibility of these changes. Ten subjects performed a 1-min maximal voluntary contraction. Activity-dependent changes in axonal excitability were measured using threshold tracking with electrical stimulation at the wrist; changes in the muscle were assessed as evoked and voluntary electromyography (EMG) and isometric force. Separate components of axonal excitability and muscle properties were tested at 5 min intervals after the sustained contraction in 5 separate sessions. The current threshold required to produce the target muscle action potential increased immediately after the contraction by 14.8% (p<0.05), reflecting decreased axonal excitability secondary to hyperpolarization. This was not correlated with the decline in amplitude of muscle force or evoked EMG. A late reversal in threshold current after the initial recovery from hyperpolarization peaked at −5.9% at ∼35 min (p<0.05). This pattern was mirrored by other indices of axonal excitability revealing a previously unreported depolarization of motor axons in the late recovery period. Measures of axonal excitability were relatively stable at rest but less so after sustained activity. The coefficient of variation (CoV) for threshold current increase was higher after activity (CoV 0.54, p<0.05) whereas changes in voluntary (CoV 0.12) and evoked twitch (CoV 0.15) force were relatively stable. These results demonstrate that activity-dependent changes in motor axon excitability are unlikely to contribute to concomitant changes in the muscle after sustained activity in healthy people. The variability in axonal excitability after sustained activity suggests that care is needed when using these measures if the integrity of either the muscle or nerve may be compromised.

## Introduction

The repetitive activity required to generate a sustained voluntary contraction produces changes in both the muscle and its motor nerve supply. In the nerve there is an activity-dependent decrease in the excitability of motor axons secondary to hyperpolarization caused by overactivity of the electrogenic Na^+^-K^+^ pump [1]–[3]. In the muscle there is a progressive decrease in its force-generating capacity [4]. There is a long-held view that the contractile and biochemical properties of a muscle are determined, at least in part, by their innervating motor axons [5] and that the properties of the motoneurones, motor units and muscles in the healthy system are tightly coupled [6]–[9]. Although sustained activity produces equivalent discharges in both the motor axons and the muscle, Ishihara and colleagues [10] concluded that chronic activity-dependent changes were more pronounced in the muscle than its nerve supply. The association between acute activity-dependent changes in nerve and muscle, their magnitude and the time course of their recovery is uncertain.

Threshold tracking has been used to examine the biophysical properties of human peripheral motor axons in vivo at rest (see [11], [12]) and in response to acute, sustained voluntary activity in healthy subjects [13]–[15]. More recently, it has been used to understand the pathophysiology of chronic disease states by examining activity-dependent changes in axonal excitability of diabetic neuropathy [16], amyotrophic lateral sclerosis [17] and chemotherapy-induced neuropathy [18]. In this technique changes in motor axon excitability are inferred from changes in the current required to generate a muscle action potential of predetermined amplitude [12] on the assumption that the measured changes in axonal excitability are reproducible and do not reflect a change in muscle properties. Similarly, activity-dependent decreases in muscle force with sustained activity are assumed to be independent of any change in excitability of the innervating motor axons [4]. These assumptions have never been directly tested.

The relationship between the properties of motoneurones, their axons and innervated muscle fibres is maintained in response to exercise [19]. The diameter of a motor axon is correlated to the electromechanical properties of the muscle fibres including contractile speed and maximal tetanic tension [20], [21]. For instance, compared with large motor axons, smaller motor axons innervate muscle fibres that produce less force, are less susceptible to fatigue, and have a greater reliance on oxidative phosphorylation [22]. This matching of properties also occurs during maximal contractions, so that declining motor axon discharge rates ‘match’ the slowing contractile speed of muscle fibres (see [4]). The transmission of impulses from the nerve to the muscle is reliable and robust because the safety margin at the neuromuscular junction ensures synaptic efficacy even during vigorous and sustained muscle activation [23]. Although action potential propagation is less secure at branch points and in terminal axons, complete block is rare except in the case of focal demyelination (e.g. [2]) and is unlikely to explain any disocciation in activity-dependent changes between the muscle and its motor innervation.

In this study we investigated whether activity-dependent reductions in the force-generating capacity of the muscle were directly associated with activity-dependent changes in motor axon excitability in healthy subjects who performed a standardised sustained voluntary task. The TROND protocol introduced in 2000 was the first semi-automated threshold tracking program (see [12]), and since then “multi-tracking” protocols have been implemented (e.g. [13]). We used the TROND protocol of threshold tracking to examine the excitability of the ulnar motor axons innervating the adductor pollicis muscle. Five parameters that could be rapidly measured were examined in detail, each of which reflected different biophysical properties of the motor axons [11], [12]. Despite its speed, multi-tracking was not used because it does not allow a detailed analysis of each parameter. In addition to measuring ulnar nerve excitability, activity-dependent changes in the adductor pollicis muscle were examined during brief maximal evoked and voluntary contractions before and after a sustained 1-min contraction sufficient to induce significant reductions in force (ie fatigue). Our aim was to investigate systematically the relationship between the changes, and the reproducibility of these changes, in both the muscle and its nerve under physiological conditions.

## Methods

Activity-dependent changes in adductor pollicis and its ulnar nerve supply were studied in the right arm of ten healthy subjects (5 men, 5 women; mean age 26 years; age range 21–42 years). All were right-handed. The study tested five specific aspects of nerve and muscle function at 5 min intervals after 1 min of sustained voluntary muscle activity. Thus each subject was tested on five separate occasions separated by at least 48 hours. Informed written consent was obtained prior to the experimental procedures which were approved by the Human Research Ethics Committee, University of New South Wales. All experiments were conducted in accordance with the Declaration of Helsinki.

### Experimental setup

**Figure 1:**
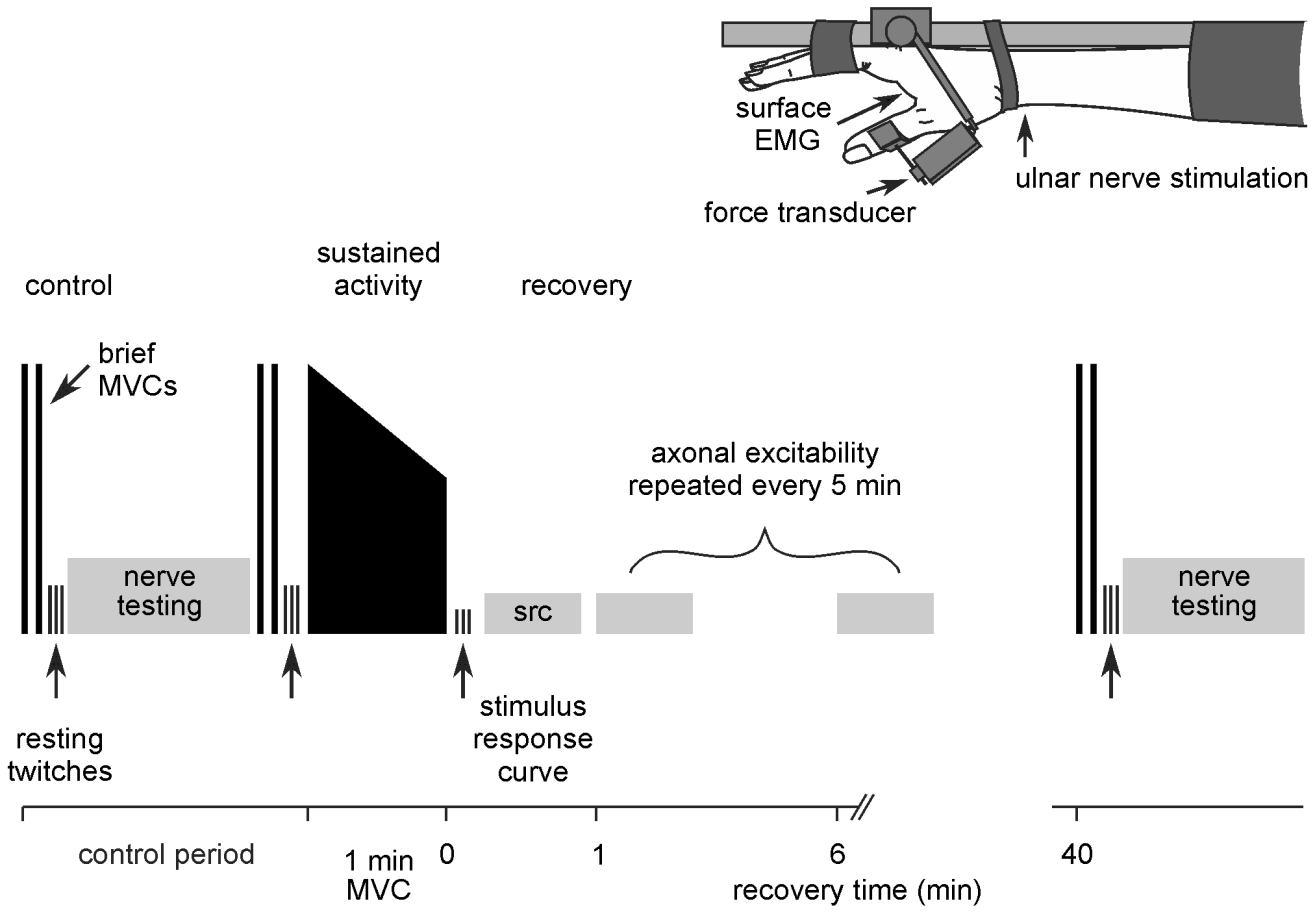
Schematic representation of the experimental setup and protocol. The experimental setup is viewed from above. The experimental protocol (not shown to scale) consisted of a control period, a sustained 1-min adductor pollicis MVC, and the recovery period. The “nerve testing” rectangle represents the TRONDNF protocol. After the stimulus-response curve (src) one component of axonal excitability was measured every 5 min for 40 min through the recovery period in 4 experimental sessions with measures of muscle force in the 5^th^ session.

Subjects sat with the test forearm supported, semi-pronated and immobilised by strapping. The wrist and digits II–V were also restrained (Fig. 1). The thumb was positioned in 75% of each subject's maximal abduction, the optimal position on the length-tension curve for adductor pollicis (McNulty PA unpublished data). Isometric adduction force was measured using a 350 N load cell (XTRAN, Applied Measurement Australia) strapped to the interphalangeal joint of the thumb. The force signal was amplified 1170 times, filtered from DC to 20 Hz (AMA2044B, Applied Measurement Australia), digitised at 2 kHz with a 1401 data acquisition card and analysed using Spike2 software (Cambridge Electronic Design, UK). Temperature was monitored at the site of nerve stimulation and maintained above 32°C using radiant heat and blankets as required.

Surface electrodes (10 mm Ag/AgCl) were used for nerve stimulation and electromyographic (EMG) recording. For nerve stimulation the cathode was positioned at the site producing an optimal muscle action potential in adductor pollicis, typically just proximal to the wrist crease and medial to the tendon of flexor carpi ulnaris. The anode was placed ∼10 cm more proximal. A switch was used to alternate between two current sources, one for testing axonal excitability and the other for testing muscle properties. For testing axonal excitability, a computer-controlled bipolar constant-current stimulator (DS5, Digitimer, UK) delivered rectangular stimuli of different pulse width, amplitude and polarity. For testing resting muscle force, rectangular pulses (0.1 ms duration) were delivered from a constant-current stimulator (DS7AH, Digitimer, UK).

EMG activity was recorded in a belly-tendon configuration with the cathode on the belly of adductor pollicis and the anode 30 mm distal. EMG signals were amplified 200 times and filtered from 10 Hz to 1 kHz (IP511, Grass, USA) with 50 Hz mains noise removed (Humbug, Quest Scientific, Canada). The EMG signal was sampled at 10 kHz (PCI-6221, National Instruments, USA) for axonal excitability testing, and at 5 kHz with a 1401 data acquisition card and Spike2 software (Cambridge Electronic Design, UK) for testing muscle properties.

### Axonal excitability testing

Activity-dependent changes in the excitability of motor axons innervating adductor pollicis were studied using a computerised threshold tracking program and the TRONDNF protocol (QTRACS, Institute of Neurology, UK). Unless otherwise stated, 1 ms test pulses were delivered at 2 Hz. Threshold tracking began with a stimulus-response curve to determine the maximum compound muscle action potential (M_max_), the amplitude of which was measured from baseline to negative peak. The target potential was set to 40% M_max_, which corresponds to the steepest point on the stimulus-response curve and is the level most commonly used in clinical studies (e.g. [18], [24]). Changes in the current required to elicit this target potential (the threshold current) were tracked throughout the protocol. A decrease in threshold current represented an increase in axonal excitability and vice versa. To confirm that changes in axonal excitability following sustained activity were due to hyperpolarization, multiple components were measured at 5 min intervals, including the strength-duration relationship, threshold electrotonus, current-threshold relationship and the recovery cycle in separate experiments. Threshold tracking was based on M_max_ rather than force output to allow for accurate measurement of all components of axonal excitability [25].

To derive the strength-duration time constant (*τ*_SD_) and rheobase, stimuli with durations of 0.2, 0.4, 0.6, 0.8 and 1.0 ms were given. For threshold electrotonus, changes in threshold current were determined at intervals from −10 to 200 ms relative to the onset of a 100 ms subthreshold conditioning pulse that was either depolarizing (+20% and +40% of threshold current) or hyperpolarizing (−20% and −40% of threshold current). The current-threshold relationship was measured by delivering a test pulse at the end of a 200 ms depolarizing or hyperpolarizing subthreshold conditioning pulse, graded in 10% steps from +50% to −100% of threshold current. for this relationship the decreases in threshold current are conventionally plotted on the x-axis while the strength of the conditioning pulses are plotted on the y-axis, despite the dependent variable being the decrease in threshold current (see [12]). The recovery cycle was used to measure changes in threshold current at 18 intervals from 2 to 200 ms after a 1 ms supramaximal stimulus. For further details of threshold tracking procedures, see Kiernan and colleagues [12] and Krishnan and colleagues [26].

### Muscle testing

To assess maximal voluntary force, subjects were instructed to contract adductor pollicis by pulling the interphalangeal joint of the thumb towards the web space between digits II–III. To ensure voluntary contractions were maximal, standardised verbal encouragement and visual force feedback were provided. Subjects were allowed to reject any contraction they considered not to be maximal. MVC force was established as the peak response during 3 brief, 2–3 s maximal efforts prior to the experimental protocol. Three supramaximal stimuli were delivered at 1 Hz immediately after the MVC to assess the twitch force of a fully potentiated muscle during relaxation. To ensure all motor axons were stimulated, the stimulus intensity was 150% of that required to produce M_max_. Pilot studies that tracked the amplitude of M_max_ throughout the protocol demonstrated that this stimulus intensity was sufficient to produce M_max_ even after 1 min of maximal voluntary activity of adductor pollicis and that the stimulus did not become submaximal in the following 40 min (see below).

### Protocol

This protocol is a modification of that used in previous studies and was designed so that changes in each measured parameter could be quantified systematically at the same constant interval following standardised voluntary activity. All subjects participated in 5 sessions so that each of four components of axonal excitability began at the same interval after the end of the sustained voluntary contraction regardless of their order in a full TRONDNF protocol. Muscle properties were tested in the same manner in the 5^th^ session. The protocol consisted of a control period in which baseline values were established, a sustained contraction, and the recovery period (Fig. 1). In the control period subjects performed 2 brief, 2–3 s MVCs immediately before 3 resting twitches were evoked. This was followed by the TRONDNF protocol, 2 brief MVCs and 3 resting twitches. In the sustained contraction subjects performed a 1-min MVC, followed immediately by 3 resting twitches. The recovery period began with a repeat of the stimulus-response curve to update the amplitude of the target potential used for threshold tracking. One parameter of axonal excitability was then measured every 5 min for 8 cycles in the first four experimental sessions. The first cycle began 1 min after the end of the MVC. In these sessions the strength-duration relationship, threshold electrotonus, current-threshold relationship and the recovery cycle were measured. Voluntary and resting twitch forces were measured in the fifth session at the same intervals. Testing continued for 40 min and finished with 2 brief MVCs, 3 resting twitches and a final TRONDNF protocol.

The amplitude of M_max_ (and consequently the target potential) was updated immediately after the 1-min MVC but not updated for another 40 min. If the size of the target potential changed markedly during this 40 min period, it may have resulted in an incorrect estimation of changes in axonal excitability. Consequently, two control experiments were performed on 5 subjects during which the amplitude of M_max_ and the target potential was updated with a stimulus-response curve every 5 min prior to the measurement of either the strength-duration relationship or the recovery cycle. Updating the amplitude and threshold current of the target potential in this way did not alter the pattern of recovery for axonal excitability.

### Data analysis

#### Measures of axonal excitability

**Figure 2:**
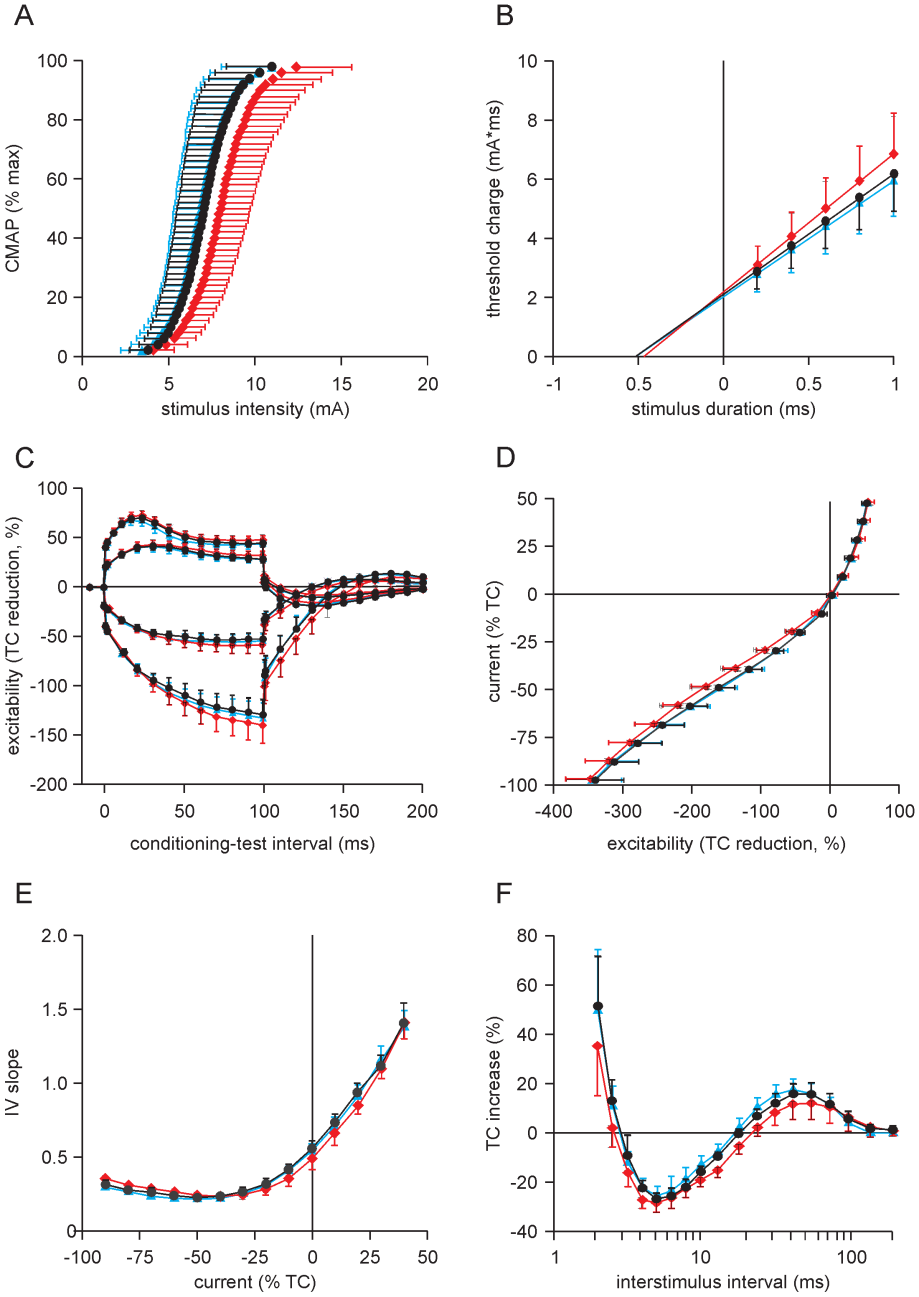
Changes in components of axonal excitability after sustained activity. Black symbols: control data; red symbols: data recorded after the 1 min sustained contraction; blue symbols: data recorded after the 40 min recovery period. A: stimulus-response curves with the CMAP normalised to M_max_. B: charge-duration curves. *τ*_SD_ and rheobase were derived from the x-intercepts and gradients of the linear regressions, respectively. C: threshold electrotonus. D: current-threshold relationship. E: slope of the current-threshold relationship. F: recovery cycle. Four subjects were excluded from the data at an interstimulus interval of 2 ms only because the output of the stimulator was insufficient to produce the target potential. Data presented as mean ± 95% confidence intervals. TC: threshold current.

Changes in the current required to generate the target potential (40% M_max_) were normalised to the threshold current value in the first stimulus-response curve. Using Weiss's law, the linear regression of threshold charge on stimulus duration was used to derive *τ*_SD_ and rheobase (see Fig. 2B). For threshold electrotonus, three time periods of interest were identified *a priori*. The first of these, S1, reflects the slow spread of subthreshold conditioning pulses to the internode (Burke *et al.*, 2001). With depolarizing currents, S1 was measured 10–20 ms after the start of the 40% depolarizing conditioning pulse (TEd^40^_10-20_). For hyperpolarizing currents, S1 was measured 10–20 ms (TEh^40^_10-20_), 20–40 ms (TEh^40^_20-40_) and 90–100 ms (TEh^40^_90-100_) after the start of the 40% hyperpolarizing conditioning pulse. S2, the second period of interest, reflects axonal accommodation to depolarizing currents (Burke *et al.*, 2001). It was measured 40–60 ms (TEd^40^_40-60_) and 90–100 ms (TEd^40^_90-100_) after the start of the 40% depolarizing conditioning pulse. Finally, the under- (TEd^40^_undershoot_) and overshoot (TEh^40^_overshoot_) in axonal excitability were measured ∼40 and ∼90 ms and after the offset of the conditioning pulses, respectively. For the current-threshold relationship, the resting IV slope was measured as the gradient between the data points for −10%, 0% and +10% conditioning pulses. Minimum IV slope was derived from the smallest gradient between any 3 adjacent data points. Hyperpolarizing IV slope was the gradient between −80%, −90% and −100% conditioning pulse data, while depolarizing IV slope was the gradient between +30%, +40% and +50% conditioning pulse data. For the recovery cycle, the durations of the refractory and superexcitable periods were extrapolated from the intercept of the curve and the x-axis of the curve (see Fig. 2D). The amplitude of superexcitability was calculated from the mean of three responses at adjacent interstimulus intervals at which the reduction in threshold current was maximal while its extent was calculated as area under the curve. The amplitude of subexcitability was calculated from the mean of three responses at adjacent interstimulus intervals beyond 10 ms at which the increase in threshold current was maximal.

#### Measures of EMG and force

The EMG signal of the sustained contraction was root mean square (RMS) processed using a 125 ms sliding window. The amplitude of EMG and force was measured as an average over 50 ms both at the start of the 1-min MVC around the absolute peak force, and immediately before the subject was instructed to relax. The resting M_max_ was measured as amplitude (baseline to negative peak and peak-to-peak) and as total area of the potential.

#### Statistical analysis

Activity-induced changes in indices of axonal excitability were assessed using one-way repeated measures ANOVA with *post-hoc* Holm-Sidak pairwise comparisons for normally distributed data, and one-way repeated measures ANOVA on ranks with *post-hoc* Tukey tests for data that were not normally distributed. Sustained activity-induced changes in muscle force, M_max_ amplitude and voluntary EMG were assessed using paired t-tests. Pearson and Spearman correlations were used for data that were normally and not normally distributed, respectively. To evaluate reproducibility, a coefficient of variation (CoV) for within-subject data was calculated. The data were normally distributed unless otherwise stated and are presented as mean and 95% confidence interval. Data that were not normally distributed are presented as median and interquartile range [IQR]. Statistical analysis was performed using SigmaStat (Systat, USA). Results were considered significant when p<0.05.

## Results

Changes in axonal excitability parameters immediately after the 1-min MVC were consistent with axonal hyperpolarisation. After the initial recovery there was a late reversal in axonal-excitability. Following maximal voluntary activity there were changes in muscle properties including resting twitch and peak voluntary force, the amplitude of M_max_, and voluntary EMG. However, none of these changes was correlated with reductions in axonal excitability. Finally, measures of axonal excitability were relatively stable at rest but less so after sustained activity.

### Changes in axonal excitability immediately after sustained activity

#### Stimulus-response curve

After the sustained maximal contraction the stimulus-response curve shifted to the right, indicating more current was required to produce the target compound muscle action potential (Fig. 2A). The mean increase in threshold current was 14.8% [9.7–20.0%] (p<0.05). This reflected a decrease in the excitability of motor axons. When the stimulus intensity was normalised to the current required to produce 50% of M_max_, the slope of the curve did not change significantly after the sustained contraction.

#### Strength-duration relationship (τ_SD_ and rheobase)

*τ*_SD_ decreased from 0.51 ms [0.47–0.55 ms] in the control period to 0.47 ms [0.44–0.49 ms] 1 min after the sustained contraction (p<0.05) (Fig. 2B). Rheobase increased from 4.1 mA [3.3–4.9 mA] to 4.7 mA [3.7–5.6 mA] (p<0.05).

#### Threshold electrotonus

Changes in threshold electrotonus after sustained activity are presented in Figure 2C. For depolarizing currents, S1 measured at TEd^40^_10-20_ (see Methods), increased from control values of 67.7% [64.2–71.2%] to 70.1% [65.8–74.4%] after the sustained contraction (p<0.05). S2, measured at TEd^40^_40-60_ and TEd^40^_90-100_, increased from 51.2% [46.4–56.0%] and 44.2% [40.0–48.4%] to 53.41% [49.1–57.8%] and 47.8% [43.2–52.4%], respectively (p<0.05). TEd^40^_undershoot_ decreased from −19.2% [−21.7–−16.7%] to −15.5% [−17.6–−13.3%] (p<0.05).

For hyperpolarizing currents, there was no significant change in TEh^40^_10-20_ after sustained activity. However, TEh^40^_20-40_ and TEh^40^_90-100_ increased from median values of −88.5% [−101.2–−85.9%] and −115.2% [−140.0–−112.8%] to −94.1% [−102.3–−89.1%] and −130.2% [−151.8–−119.7%], respectively (p<0.05). TEh^40^_overshoot_ decreased from 13.9% [12.1–15.6%] to 9.1% [5.7–12.4%] (p<0.05).

#### Current-threshold relationship

The sustained contraction produced changes in the current-threshold relationship (Fig. 2D). Changes in the slope of the IV curve are illustrated in Figure 2E. After the sustained contraction resting IV slope decreased from a control value of 0.56 [0.50–0.62] to 0.49 [0.42–0.56] (p<0.05). There was no significant change in the depolarizing, hyperpolarizing or minimum IV slopes.

#### Recovery cycle

The duration of the refractory period decreased from 2.9 ms [2.6–3.1 ms] to 2.6 ms [2.4–2.8 ms] after the sustained contraction (p<0.05) (Fig. 2F). The amplitude of the relative refractory period measured at 2.5 ms decreased from 12.9% [4.0–21.9%] in the control period to 2.1% [−5.7–9.9%] (p<0.05). There was no significant change in the amplitude of superexcitability after the 1-min MVC, but its duration and area increased. The duration increased from 15.8 ms [14.1–17.6 ms] to 20.3 ms [16.4–24.2 ms] while the area increased by 32.7% [18.4–47.0%] (p<0.05). The amplitude of subexcitability did not change significantly.

### Time course of recovery in axonal excitability

**Figure 3:**
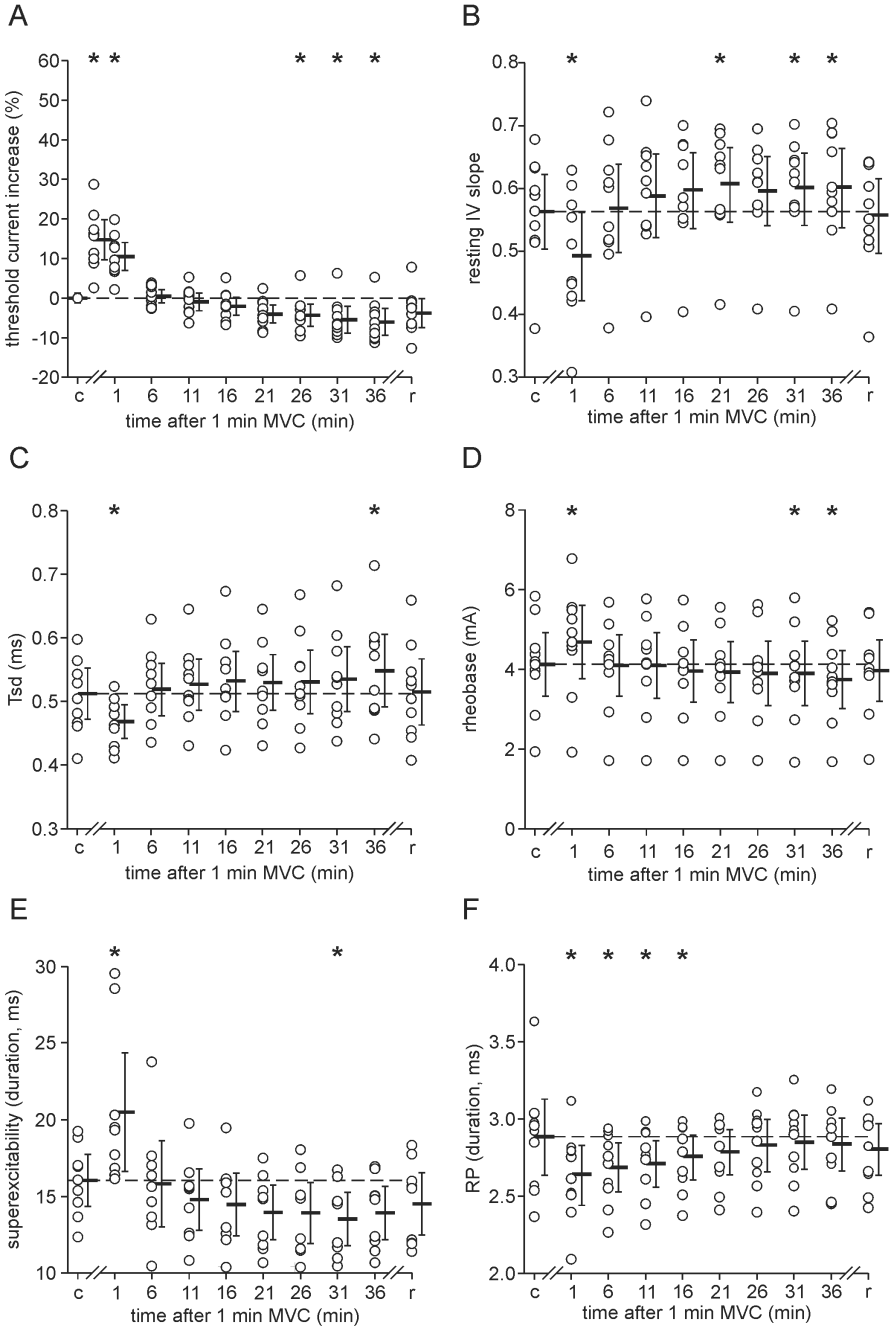
Recovery of axonal excitability after sustained activity. Open symbols represent the data for each subject; pooled data are presented as mean (solid bars) and 95% confidence intervals. A: threshold current. B: resting IV slope. C: *τ*_SD_. D: rheobase. D: the duration of the superexcitable period. F: the duration of the refractory period (RP). *: p<0.05.

Threshold current returned to control values by 6 min (Fig. 3A). Other indices that had recovered by 6 min were *τ*_SD_, rheobase, TEd^40^_10-20_, TEd^40^_40-60_, TEd^40^_90-100_, TEh^40^_20-40_, TEh^40^_90-100_, resting IV slope, and the duration and area of superexcitability. Indices with slower time courses of recovery were (i) the magnitude and duration of the relative refractory period, which returned to control values by 21 min (Fig. 3F) and (ii) TEd^40^_undershoot_, which had not returned to control values by 40 min.

After the initial recovery period threshold current showed a late reversal which was significant at 26 min [−4.2%; −7.2–−1.3%], 31 min [−5.2%; −8.7–−1.8%] and 36 min [−5.9%; −9.4–−2.3%] (p<0.05) (Fig. 3A). This late reversal also occurred for resting IV slope at 21, 31 and 36 min (Fig. 3B); *τ*_SD_ at 31 min (Fig. 3C); rheobase at 31 and 36 min (Fig. 3D); the duration of superexcitability at 31 min (Fig. 3E); and the area of superexcitability at 26, 31 and 36 min (p<0.05).

### Changes in M_max_, voluntary EMG, twitch force and maximal voluntary force

**Figure 4:**
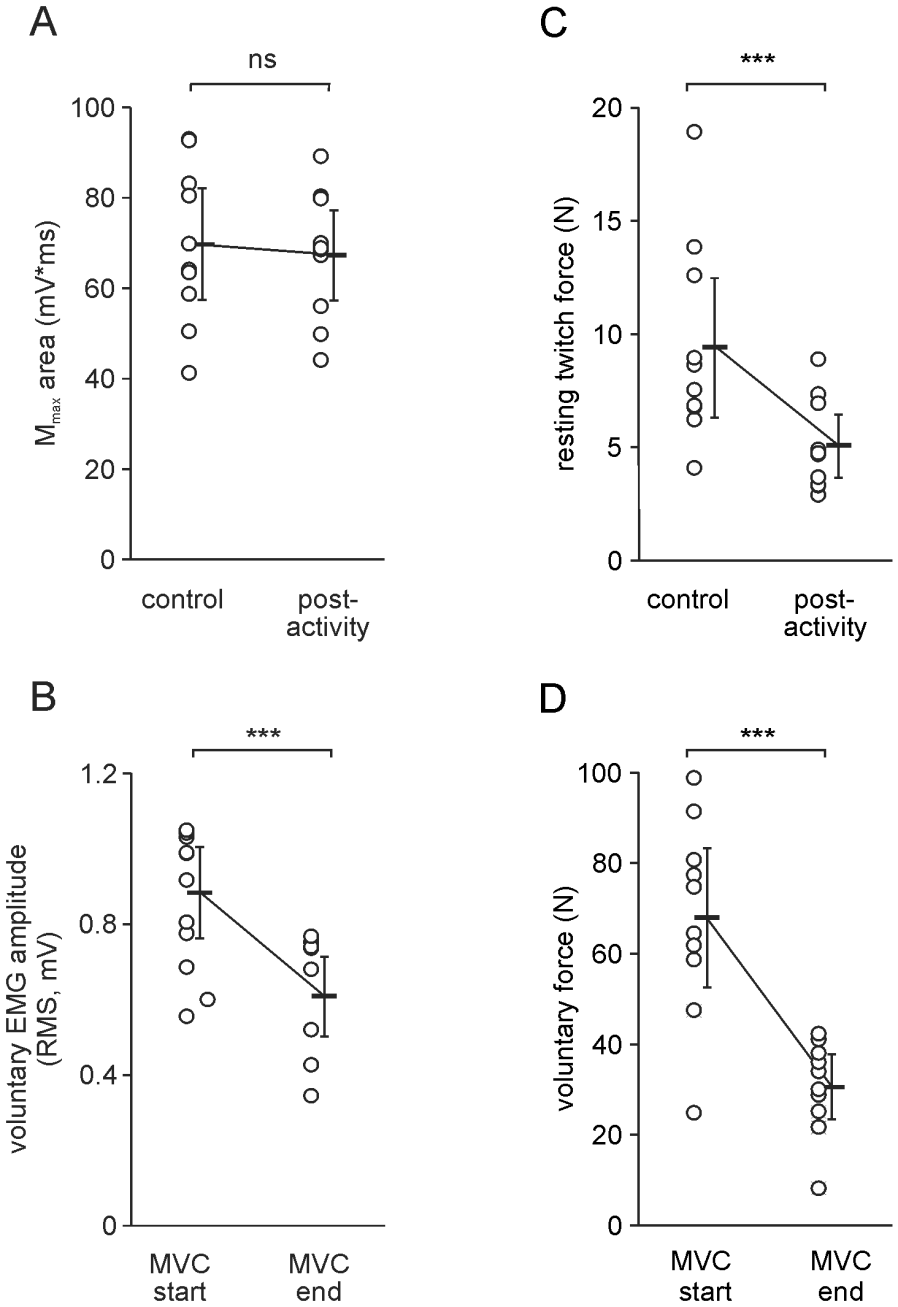
Changes in muscle properties with sustained activity. Open symbols represent the mean for each subject across 5 experimental sessions; pooled data for 10 subjects are shown beside the individual data as mean (solid bars) and 95% confidence intervals. A: there was no change in the area of M_max_ after the sustained contraction; B: there was a significant change in the resting twitch force immediately after the 1-min MVC compared to control values; C: the peak amplitude of the voluntary EMG was significantly larger at the start than at the end of the 1-min MVC; D: peak voluntary force was significantly smaller at the end of the 1-min MVC than at the start demonstrating an activity-dependent reduction in the force-generating capacity of the muscle (ie fatigue). ns: not significant; ***: p<0.001.

There was a significant decrease in the (updated) peak amplitude of M_max_ after the sustained contraction. The baseline to negative-peak amplitude decreased from 11.1 mV [9.6–12.6 mV] to 9.7 mV [8.4–11.0 mV] and the peak-to-peak amplitude decreased from 19.1 mV [16.3–22.0 mV] to 17.2 mV [14.7–19.7 mV] (p<0.001). However, the area of M_max_ did not change significantly (Fig. 4A). At the start of the 1-min MVC the amplitude of voluntary EMG was 0.9 mV (RMS) [0.8–.0 mV]. This decreased to 0.6 mV [0.5–0.7 mV] at the end of the contraction (p<0.001; Fig. 4B).

Resting twitch force decreased by 42% after the sustained contraction, from 9.5 N [6.3–12.6 N] before the 1–min MVC to 5.1 N [3.7–6.5 N] (p<0.001) after the 1-min MVC (Fig. 4C). Peak voluntary force was 68.3 N [52.9–83.7 N] at the start of the 1-min MVC and this decreased to 30.9 N [23.5–38.3 N] at the end of the contraction (p<0.001), a decline of 55% (Fig. 4D).

### Relationship between changes in axonal and muscle properties

**Figure 5:**
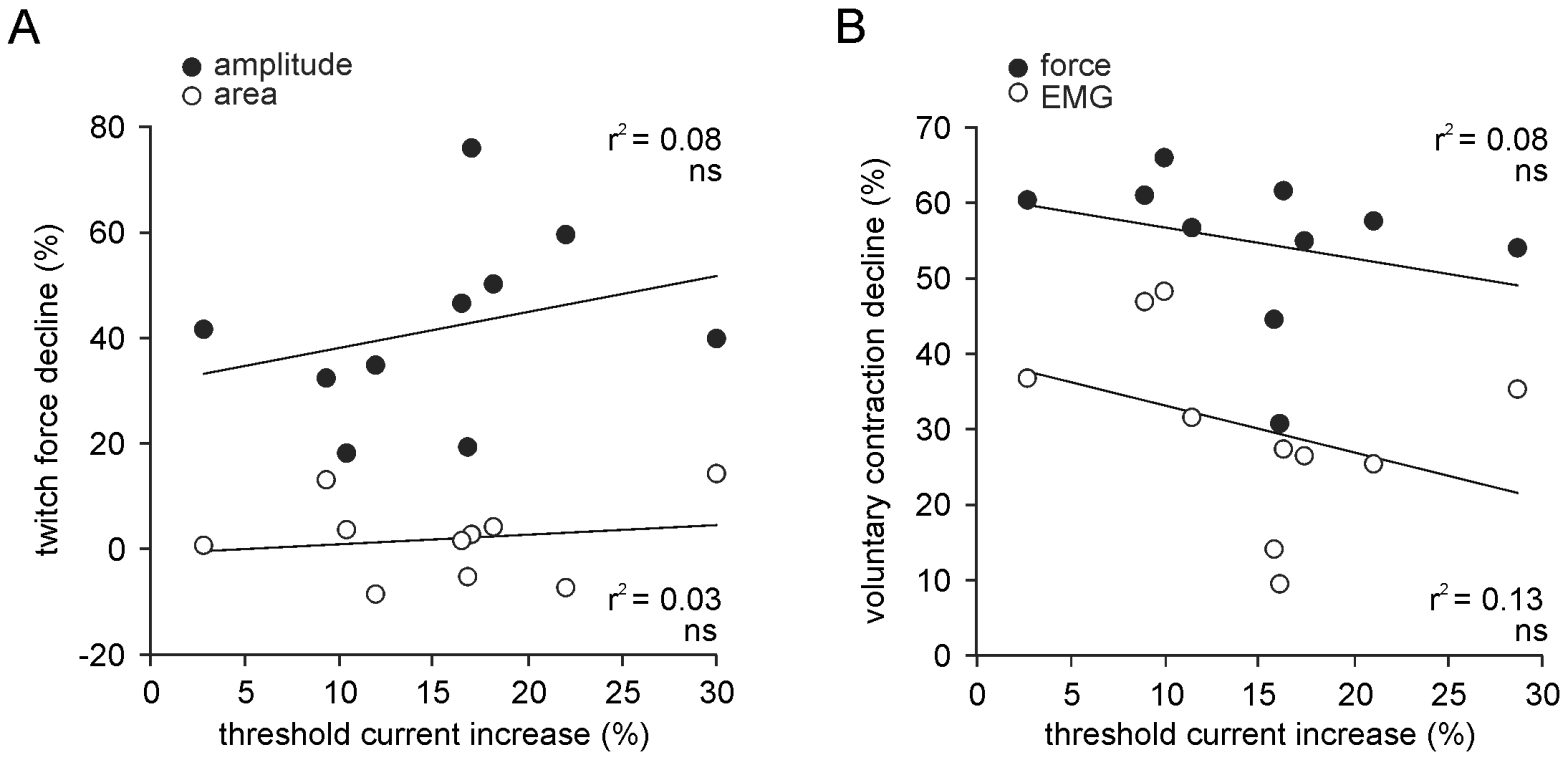
Relationship between the activity-dependent changes in muscle output and axonal excitability. Symbols represent the mean for each subject across 5 experimental sessions. A: There was no relationship between the activity-dependent increases in threshold current and the decline in the amplitude (black symbols) or area (white symbols) of resting twitch force. B: Similarly, there was no relationship between increases in threshold current and the decline in voluntary force (black symbols) and voluntary EMG (white s). ns: not significant.

There were no significant correlations between the decrease in motor axonal excitability measured as the increase in the threshold current required to produce 40% of M_max_ in the updated stimulus-response curve, and the decline in the area or amplitude of the twitch force in the relaxed muscle (Fig 5A). Importantly, there was no significant correlation between decreased motor axon excitability and the decline in maximal voluntary muscle output, whether measured as EMG or force (Fig. 5B).

### Intersession variability

The repeated testing protocol allowed for the assessment of inter-session variability. Some measures were relatively stable between sessions including the majority of axonal excitability indices at rest, such as threshold current measured before the 1 min-MVC [CoV (median and IQR) 0.12; 0.06–0.18]; and the extent of voluntary force production, measured as the force decline during the 1-min MVC [CoV 0.12; 0.06–0.18]. The decline in resting twitch force after the 1-min MVC was of was slightly more variable [CoV 0.15; 0.23–0.44]. The increase in threshold current following sustained activity was most variable [CoV 0.54; 0.33–0.65], and its coefficient of variation was significantly higher than that for threshold current at rest or the reduction in force during the 1-min MVC (p<0.05).

## Discussion

In this study we systematically investigated the relationship between activity-dependent changes in the adductor pollicis muscle and the motor axons of its ulnar nerve supply. Our key finding was that the changes in the adductor pollicis muscle were independent of the activity-dependent changes in the motor axons despite the same high demand on both the muscle and its nerve supply. This suggests that decreased axonal excitability makes no contribution to the reduction in the force-generating capacity of the muscle that occurs as a consequence of sustained voluntary activity, ie fatigue. This is the first study to examine the reproducibility of activity-dependent changes in motor axons and muscles. We found that the observed increase in threshold current was the least reproducible parameter studied, and that measures of axonal excitability are significantly more variable after voluntary muscle activity than at rest or for any measure of muscle output. A novel observation was a reversal in the excitability of the motor axons, most noticeably of threshold current, that occurred late in recovery period and presumably reflects the delayed deactivation of currents opposing hyperpolarization. These results provide a note of caution when using axonal excitability threshold tracking in diseases that may affect the muscle and nerve differently. In such cases a change in the properties of the muscle cannot necessarily be used as the basis for inferring a change in the properties of the motor axon membrane.

### Decreased motor axonal excitability reflected hyperpolarization

The pattern of changes in multiple indices of axon function suggests that decreased axonal excitability with sustained activity was caused by hyperpolarization. Specifically, *τ*_SD_ declined and rheobase increased, consistent with a hyperpolarization-induced reduction in the persistent Na^+^ current (*I*_NaP_) at the node [27], [28]. The increased absolute magnitude of the threshold electrotonus, S1 and S2 produced a ‘fanned out’ appearance [29], reflecting increased resistance of the internodal membrane due to a hyperpolarization-mediated reduction in K^+^ channel activation [11]. In contrast, both TEd^40^_undershoot_ and TEh^40^_overshoot_ decreased in absolute magnitude, resulting in a ‘fanned in’ appearance. Resting IV slope decreased, suggesting a decreased input conductance of the axon secondary to hyperpolarization [30]. The decrease in relative refractory period suggests a reduction in Na^+^ channel inactivation [11]. The duration and area of superexcitability increased, presumably due to an augmented depolarizing afterpotential secondary to a hyperpolarization-mediated inactivation of K^+^ channels with fast kinetics (K_f_) [31]. Overall, these changes reflected nodal and intermodal hyperpolarization.

There is a discrepancy between the findings on axonal changes of the present study and the results of Kuwabara and colleagues [13]. In the latter study the authors reported an increase in the threshold current of the relative refractory period (measured at 2 ms) following a sustained contraction lasting ∼3 min despite having expected a decline because of hyperpolarization. The amplitude of M_max_ was decreased when evoked by closely spaced (2–4 ms) paired pulses, which was proposed to reflect activity-dependent conduction block at distal axon branch points and nerve terminals [13], shown to be sites of lowered safety margin [32]–[34]. In the present study there was no evidence of a transient increase in the refractory period. Kuwabara and colleagues [13] measured activity-dependent changes in the recovery cycle at only two interstimulus intervals within the refractory (2 ms) and superexcitable (7 ms) periods. In contrast, the recovery cycle test used here as part of the TRONDNF protocol, worked backwards from 200 to 2 ms, taking ∼2 min to “reach” the relative refractory period. This delay may contribute to the difference in findings between the present study and that of Kuwabara and colleagues [13]. However, the physiological relevance of any conduction block with 2–4 ms interstimulus intervals is uncertain because they correspond to motoneurone firing rates of ∼250–500 Hz, which are rarely encountered in human motor tasks [35], except briefly in ballistic efforts [36] or with doublet discharges [37].

### Late reversal in axonal excitability

A late reversal in threshold current followed the initial recovery from hyperpolarization. This pattern was mirrored by other indices of axonal excitability including *τ*_SD_ and superexcitability, suggesting the reversal in threshold current reflected a subsequent depolarization. This late period of axonal depolarization has not previously been reported following voluntary activity, perhaps because recovery has not been followed for >20 min. Kiernan and colleagues [38] measured the recovery of median motor axons for ∼35 min after 10 min of 8 Hz electrical stimulation. Although not noted by the authors, a late reversal in threshold current as observed here is evident in their Figure 1A. Similar phenomena have been reported in animal studies. Specifically, depolarizing reversals in membrane potential occurred in motoneurones of the cat [39] and sensory ganglion neurones of the mouse [40] following hyperpolarizing pulses, albeit over much shorter time courses (∼200 ms). Presumably these reversals were mediated by the delayed deactivation of a hyperpolarization-activated cation current known as *I*_h_ [40], an internodal rectifying channel that counters hyperpolarization by permitting a net inward cationic current [26], [41].

We know of no animal studies that applied hyperpolarizing pulses of very long duration (minutes) to motor axons; nor has this been done in human studies due to the discomfort involved. Nevertheless, we propose that the late reversal in threshold current in this study reflected delayed *I*_h_ deactivation as changes in axonal excitability after voluntary activity represent a balance between the opposing actions on membrane potential of the Na^+^-K^+^ pump [1] and *I*_h_ [40]–[42]. Immediately after sustained activity, the electrogenic effects of the Na^+^-K^+^ pump predominate, resulting in hyperpolarization. Later in the recovery period when the Na^+^-K^+^ pump is less active, the effects of *I*_h_ may predominate, particularly given its slow kinetics of deactivation [43], resulting in a net depolarization of the motor axon [44].

Repeated axonal excitability testing is unlikely to have contributed to this delayed depolarisation. A study by Howells and colleagues [45] demonstrated that measures of axonal excitability are highly reproducible within a subject during the same experimental session, despite the session involving repeated stimuli and multiple excitability protocols. The stability of threshold tracking over a prolonged time period (6 hours) in the absence of activity has also been demonstrated [46].

### Magnitude of activity-dependent changes

The decline in muscle force after the sustained contraction in the present study was similar to previous reports of ∼50% (e.g. [47]). The reported decrease in axonal excitability in median motor axons has been more variable, ranging from ∼38% [14], [15] to∼15% [48]. The decline of 14.8% in the present study is similar to the decline of 18.3% in a previous study of 20 subjects after a 1-min MVC of adductor pollicis [49]. This smaller decline in excitability may reflect different experimental protocols. Measurement of the stimulus-response curve in the present study was completed after the sustained contraction and took ∼30 s. This unavoidable delay may have produced an underestimation of excitability changes. However, the updated stimulus-response curve was critical to adjust for sustained activity-induced changes in the amplitude of the target muscle action potential (see above).

The magnitude of axonal excitability changes may also reflect the nerve and muscle tested. Previous studies examined median motor axons innervating abductor pollicis brevis [13], [15]. Changes in the present study are consistent with the only other study to have examined sustained activity of ulnar motor axons innervating adductor pollicis [49]. Differences in biophysical properties have been reported at rest for ulnar and median motor axons [50], and perhaps these differences affect the magnitude of hyperpolarization following sustained activity. Specifically, the change in threshold electrotonus in the hyperpolarizing direction was smaller in ulnar motor axons than in median motor axons [50]. This was interpreted to reflect a difference in the activity of *I*_h_, with respect to either the specific isoform of the HCN channel or threshold for activation [50]. It is possible that this limits the extent of hyperpolarisation in ulnar motor axons, and produces a more prominent late reversal in axonal excitability.

It is difficult to compare the magnitude of these excitability changes to those in disease states because of the choice of nerve and muscle. Previous studies of neuropathic nerves were conducted using the median nerve and abductor pollicis brevis muscle (e.g. [16], [17]) rather than the ulnar nerve and adductor pollicis of this study. Not all neuropathies are associated with a change in the extent of activity-dependent hyperpolarisation. For example, for patients with end-stage kidney disease there was an 18.4% decrease in axonal excitability after a 1-min MVC [48], but this was not significantly different to healthy controls (15.0%) or this study. For neuropathies associated with a substantial change in the extent of hyperpolarisation there is significant variation according to disease state. Patients with diabetic neuropathy had a small decrease in axonal excitability (13.1%), thought to reflect dysfunction of the Na^+^-K^+^ pump [16], while patients with amyotrophic lateral sclerosis had a larger increase (36.5%), thought to be due to higher discharge rates of the surviving motor axons [17].

### Reproducibility

Variability of axonal excitability indices at rest have been studied in motor axons of the median [46] and ulnar nerves [50]. However, this is the first study to report the reproducibility of decreases in axonal excitability after sustained activity. After sustained activity the increase in threshold current (reflecting decreased axonal excitability) was the most variable within-subjects measure. This was despite the reproducibility of reductions in voluntary force during the 1-min MVC, which was facilitated by the standardisation of the MVC in this study, unlike previous reports. This suggests the extent of axonal hyperpolarization with sustained voluntary contractions is intrinsically variable. This variability may be related to day-to-day fluctuations in factors affecting Na^+^-K^+^ pump activity, including resting membrane potential [51], and electrolyte status [52].

### Relationship between changes in motor axonal excitability and properties of the muscle

This is the first study to assess the correlation between decreases in motor axonal excitability and muscle output, such as correlated reductions in axonal excitability and resting twitch force. No correlation was observed, so that activity-dependent changes in axonal excitability were not correlated with activity-dependent changes in adductor pollicis despite the widespread assumption that the properties of motoneurones, their axons and the innervated muscle fibres are closely matched [6]–[9].This finding also suggests that muscle force production is not compromised by activity-induced axonal hyperpolarization in healthy subjects, presumably due to the large safety margin for saltatory conduction [53], [54] and at the neuromuscular junction [23]. Conduction block predominantly occurs in disease states with localised demyelinating neuropathies [55], [56].

In conclusion, we have shown that the activity-dependent changes that occur in the muscle after sustained activity follow different time courses to, and are not correlated with, changes that occur in its motor nerve supply. In the healthy population such changes do not impede impulse conduction, even after intense but brief maximal voluntary activity. These results provide confidence that the safety margin of both the motor axons and the neuromuscular junction is more than sufficient to accommodate sudden increases in sustained activity. Our data suggest that although these methods are robust in healthy adults, care is required when interpreting activity-dependent neuromuscular excitability changes with pathologies.
